# A Neuroprotective Bovine Colostrum Attenuates Apoptosis in Dexamethasone-Treated MC3T3-E1 Osteoblastic Cells

**DOI:** 10.3390/ijms221910195

**Published:** 2021-09-22

**Authors:** Sagrario Martin-Aragon, Paloma Bermejo-Bescós, Juana Benedí, Carlos Raposo, Franklim Marques, Eirini K. Kydonaki, Paraskevi Gkiata, Yiannis Koutedakis, Georgia Ntina, Andres E. Carrillo, Tânia Amorim

**Affiliations:** 1 Department of Pharmacology, Pharmacognosy and Botany, Complutense University, 28040 Madrid, Spain; smartina@ucm.es (S.M.-A.); bescos@ucm.es (P.B.-B.); jbenedi@ucm.es (J.B.); craposoc@gmail.com (C.R.); 2 SALURIS, 28040 Madrid, Spain; 3 UCIBIO/REQUIMTE, Faculty of Pharmacy, University of Porto, 4099-002 Porto, Portugal; franklim@ff.up.pt (F.M.); eir.kyd@gmail.com (E.K.K.); 4 School of Sport and Exercise Sciences, University of Thessaly, Karies, 42100 Trikala, Greece; gkiata.vivi@gmail.com (P.G.); y.koutedakis@uth.gr (Y.K.); 5 Faculty of Education, Health and Wellbeing, Wolverhampton University, Walsall WV1 1LY, UK; 6 BME, Biomechanical Solutions, 43150 Karditsa, Greece; ntinageorgia10@gmail.com; 7 Department of Exercise Science, Chatham University, Pittsburgh, PA 15232, USA; acarrillo@chatham.edu; 8 Move-Cor Inc., Pittsburgh, PA 15017, USA

**Keywords:** bovine colostrum, neuroprotective, osteoblast, glucocorticoid-induced osteoporosis, dexamethasone, ERK1/2, caspase-3, glutathione reduced, Hsp70

## Abstract

Glucocorticoid-induced osteoporosis (GIO) is one of the most common secondary forms of osteoporosis. GIO is partially due to the apoptosis of osteoblasts and osteocytes. In addition, high doses of dexamethasone (DEX), a synthetic glucocorticoid receptor agonist, induces neurodegeneration by initiating inflammatory processes leading to neural apoptosis. Here, a neuroprotective bovine colostrum against glucocorticoid-induced neuronal damage was investigated for its anti-apoptotic activity in glucocorticoid-treated MC3T3-E1 osteoblastic cells. A model of apoptotic osteoblastic cells was developed by exposing MC3T3-E1 cells to DEX (0–700 μM). Colostrum co-treated with DEX was executed at 0.1–5.0 mg/mL. Cell viability was measured for all treatment schedules. Caspase-3 activation was assessed to determine both osteoblast apoptosis under DEX exposure and its potential prevention by colostrum co-treatment. Glutathione reduced (GSH) was measured to determine whether DEX-mediated oxidative stress-driven apoptosis is alleviated by colostrum co-treatment. Western blot was performed to determine the levels of p-ERK1/2, Bcl-XL, Bax, and Hsp70 proteins upon DEX or DEX plus colostrum exposure. Colostrum prevented the decrease in cell viability and the increase in caspase-3 activation and oxidative stress caused by DEX exposure. Cells, upon colostrum co-treated with DEX, exhibited higher levels of p-ERK1/2 and lower levels of Bcl-XL, Bax, and Hsp70. Our data support the notion that colostrum may be able to reduce DEX-induced apoptosis possibly via the activation of the ERK pathway and modulation of the Hsp70 system. We provided preliminary evidence on how bovine colostrum, as a complex and multi-component dairy product, in addition to its neuroprotective action, may affect osteoblastic cell survival undergoing apoptosis.

## 1. Introduction

Osteoporosis is a systemic degenerative disease during aging which is associated with increased fragility and fracture risk of bone [1]. In particular, osteoporotic fractures are most common in postmenopausal women or elderly people, thereby significantly affecting their life quality and expectancy [2]. Younger populations may also demonstrate increased fragility and fracture risk of bone [3,4]. In addition, there is a strong correlation between cognitive impairment and bone diseases. Impaired cognitive functions and neurodegeneration are often associated with defects in bone [5], while therapeutic strategies aiming to improve bone status are associated with a lower risk of cognitive impairment or dementia [6]. Particularly, it has been shown that low bone density and osteoporosis may be related to dementia and Alzheimer’s disease (AD) in postmenopausal women [7,8]. Moreover, glucocorticoid hypersecretion has various adverse effects including inhibiting neurogenesis and impairing the ability of neurons to survive. Moreover, glucocorticoids, used as a treatment of chronic diseases based on their anti-inflammatory, immune-modulatory, and antiproliferative properties, are associated with serious side effects, including osteoporosis [9]. In fact, glucocorticoid-induced osteoporosis (GIO) is one of the most common secondary and iatrogenic forms of osteoporosis and the resulting fractures cause significant morbidity [10]. Furthermore, it has been proven that a high dose of dexamethasone induces neurodegeneration by initiating inflammatory processes that lead to neural apoptosis. The toxicity of dexamethasone appears to be based not only on its toxic effect on neuronal function, but also on impairment of the nervous system leading to neuronal death processes [11].

On the other hand, milk and dairy product supplementation is widely recommended to prevent osteoporosis and subsequent fractures, as selected for their functional ingredients, including milk basic protein, casein phosphopeptide, and lactoferrin, are beneficial for bone health [12]. For instance, colostrum is the first nourishment of mammalian neonates containing nutrient-rich, immune, developmental, and tissue-repairing factors produced by the mammary glands shortly after birth [13]. Particularly, several studies have shown that colostrum is an important functional substance for bone health [14] and cognitive function [15]. However, the high amount of potentially active compounds in colostrum composition makes it difficult to know which of them are responsible for the potential health benefits. Despite this constraint, the vast majority of colostrum constituents have been tested singly on numerous cell-based systems and, particularly, there is ample knowledge of their antioxidant, anti-apoptotic, and cell proliferation/differentiation-promoting activities. Antioxidant components of colostrum, such as the iron-binding protein lactoferrin, the colostral component similar to ascorbate [16], glutathione peroxidase, catalase [17], and proline-rich polypeptide (PRP) complex known as colostrinin [18,19], among others, have been shown to prevent oxidative cell damage both in osteoblast and neurons. Furthermore, in vitro modulation of cell proliferation/differentiation and apoptosis has been described by colostrum constituents such as ribonucleosides [20], colostrinin [21], insulin-like growth factor 1 (IGF-1) [22,23], growth hormone (GH), platelet-derived growth factor (PDGF) [24], insulin [25], leptin [26], unsaturated fatty acids and calcium [27] and lactoferrin [28].

Regarding the great concern raised on GIO, it is worth studying in-depth its underlying mechanism and strategies to overcome it. In this context, it is widely described that apoptosis of osteoblasts and osteocytes as well as prolongation of osteoclast lifespan are the principal pathogenesis of GIO [29]. Oxidative stress induced by glucocorticoids could also promote apoptosis of the osteoblasts [30]. The increased production of reactive oxygen species (ROS) is considered the primary mechanism of the inhibition of osteoblastic proliferation [31]. Hence, preventing osteoblast apoptosis might suppress the development of GIO and might be a promising therapeutic strategy for the treatment of GIO. 

Interestingly, in our lab screen for the search of neuroprotective compounds and products, bovine colostrum displayed some degree of neuroprotective activity when tested in a glucocorticoid (GC)-induced neuroblastoma cell model of damage. Based on this finding and the remarkable composition of bovine colostrum and the activities of its components reported in the literature [16,17,18,19,20,21,22,23,24,25,26,27,28], we proceeded our study focusing on the effects of this particular colostrum on dexamethasone (DEX)-treated MC3T3-E1 osteoblastic cells for assessing a possible anti-apoptotic activity. DEX has been chosen, among the various GCs, on the basis that phenotypic changes derived from cell exposure to this GC are more consistent and prominent [32,33]. 

In this way, we aim to hypothesize whether the present bovine colostrum possesses potential as a natural resource for the prevention and/or treatment of the two closely linked multifactorial progressive degenerative disorders, osteoporosis, and neurodegenerative pathologies. 

## 2. Results

### 2.1. Cell Model of Neurotoxicity by SH-SY5Y Cell Exposure to Dexamethasone (DEX)

**Figure 1:**
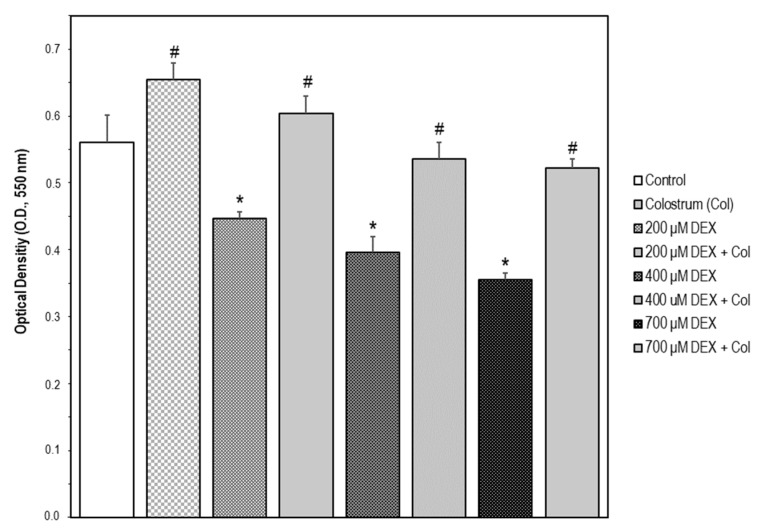
Cell model of neurotoxicity by SH-SY5Y cell exposure to dexamethasone (DEX). Cell viability was measured by the MTT assay. The data are expressed as the mean ± S.E.M. of three independent experiments. A set of SH-SY5Y cells were treated with DEX in a concentration range of 0–700 μM for 48 h. Significant differences were found in optical density (OD, 550 nm) values elicited by every DEX concentration compared to the control untreated cells (Newman–Keuls test, * *p* < 0.05 vs. control). Another set of cells were treated with colostrum (1.0 mg/mL) for 1 h and then cell incubation continued with DEX (200, 400, or 700 μM) for 48 h. Significant differences were found in OD values elicited by colostrum alone compared to the control untreated cells (Newman–Keuls test, ^#^ *p* < 0.05). Significant differences of DEX plus colostrum versus DEX-treated cells were found at any DEX concentration (Newman–Keuls test, ^#^ *p* < 0.05).

The cellular model for neuronal damage we have chosen was the SH-SY5Y human neuroblastoma cell line as it has been developed earlier, proving that a high dosage of DEX is neurotoxic through impairment of mitochondrial dynamics [11]. Therefore, SH-SY5Y cells were treated with DEX in a concentration range of 0–700 μM for 24 and 48 h, respectively. In our experimental model of SH-SY5Y cells, there was no observable increase in cell death following DEX treatment up to 24 h. However, a certain lowering of cell viability using the MTT assay was observed from 200 to 700 μM for 48 h (Figure 1).

### 2.2. Neuroprotective Effect of Colostrum on DEX-Induced SH-SY5Y Cell Damage

**Figure 2:**
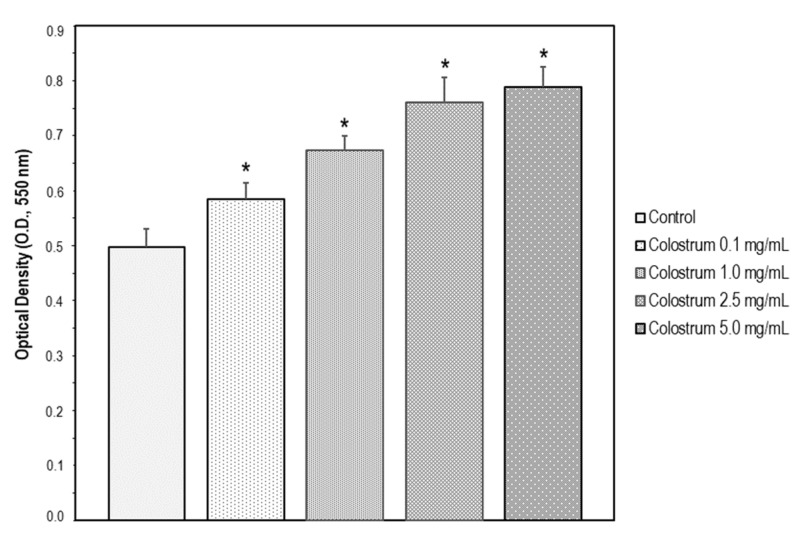
SH-SY5Y cells were treated with Colostrum (0.1, 1.0, 2.5 and 5.0 mg/mL) for 24 h. Then, cell viability was measured by the MTT assay. The data are expressed as the mean ± S.E.M. of three independent experiments. Significant differences were found in OD values elicited by every Colostrum concentration compared to the control untreated cells (Newman–Keuls test, * *p* < 0.001 vs. control).

The bovine colostrum was assayed at different concentrations (0.1, 1.0, 2.5, and 5.0 mg/mL) in SH-SY5Y cells for 24 h. The increase in cell viability upon colostrum was concentration-dependent (Figure 2). To investigate whether the bovine colostrum may protect SH-SY5Y cells against DEX-induced neurotoxicity, the cells were treated with 1.0 mg/mL colostrum for 1 h. Then, DEX was added to the culture media at 200, 400, and 700 μM, respectively, and cell incubation continued for 48 h. Afterwards, cell viability was examined, and it was found that colostrum was capable of rescuing cells in the presence of any concentration of DEX (Figure 1).

### 2.3. ROS Scavenger Activity of Bovine Colostrum in SH-SY5Y Cells

**Figure 3:**
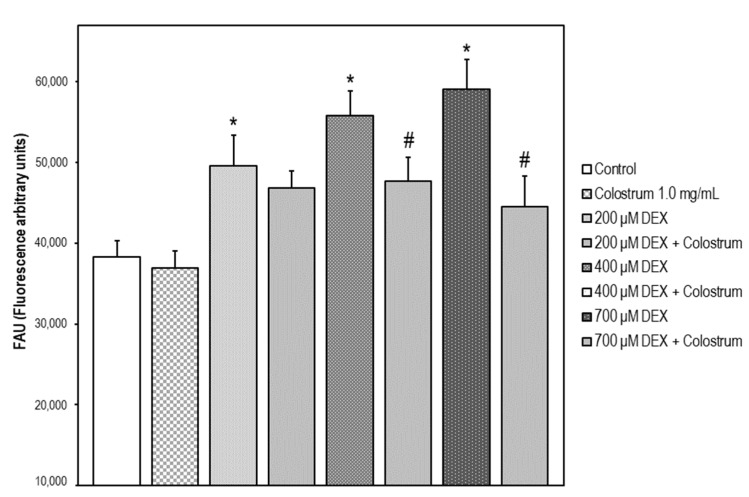
ROS production (DCFH-DA test) in SH-SY5Y cells after 48 h treatment with DEX. Data are given as the mean of fluorescence units ± S.E.M. of three separate experiments. The data are expressed as the mean ± S.E.M. of three independent experiments. Fluorescence was measured at λexc = 485 nm (bandwidth 5 nm) and λem = 520 nm (bandwidth 5 nm). A set of SH-SY5Y cells were treated with DEX in a concentration range of 0–700 μM for 48 h. Significant differences were found in fluorescence intensity values elicited by every DEX concentration compared to the control untreated cells (Newman–Keuls test, * *p* < 0.05 vs. control). Another set of cells were treated with colostrum (1.0 mg/mL) for 1 h and then cell incubation continued with DEX (200, 400, or 700 μM) for 48 h. Significant differences of DEX plus colostrum versus DEX-treated cells were found at 400 and 700 μM DEX concentrations (Newman–Keuls test, ^#^ *p* < 0.05).

Exposure of the cells to 200–700 μM DEX μM for 48 h caused a 30, 46, and 54% increase in fluorescence intensity, respectively, relative to DEX-untreated control cells. A 48 h treatment of the cells with colostrum at a concentration of 1.0 mg/mL plus DEX diminished the fluorescence intensity by 21 and 38%, respectively, in comparison with the cells exposed to 400 and 700 μM DEX. No significant changes in fluorescence intensity versus control cells were detected in non-stressed cells treated only with the bovine colostrum (Figure 3).

### 2.4. DEX-Induced Caspase-3 Activation SH-5Y5Y Is Attenuated by Colostrum Treatment

**Figure 4:**
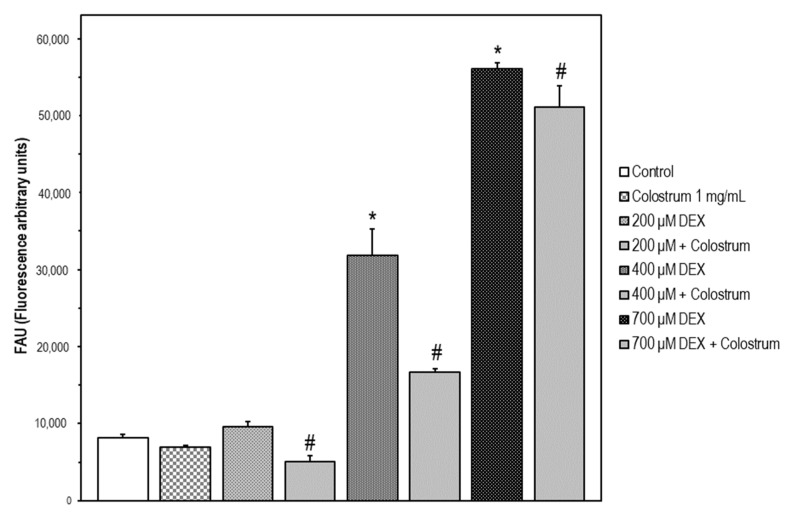
DEX-induced caspase-3 activation in SH-5Y5Y is attenuated by colostrum treatment. Caspase-3 activity was determined using the fluorogenic substrate Ac-DEVD-AMC. Data are expressed in fluorescence arbitrary units (FAU) and represent the mean ± S.E.M. of three independent experiments. Significant differences of 400 μM and 700 μM DEX treated cells versus the control untreated cells were found (Newman–Keuls test, * *p* < 0.001 vs. control). Significant differences of colostrum plus DEX treated cells versus DEX-treated cells were found (Newman–Keuls test, ^#^ *p* < 0.05 vs. DEX treated cells).

In our experiments, 200–700 μM DEX treatment for 48 h rendered a significant increase in caspase-3 activation versus untreated SH-SY5Y cells. The colostrum alone did not affect caspase-3 activation. Our results indicated that DEX induces caspase-3 activation, as a key mediator of cell death, rather than reducing proliferation. For any DEX concentration, colostrum was capable of attenuating caspase-3 activation when cells were treated with colostrum at 1.0 mg/mL in co-treatment with DEX for 48 h (Figure 4). The highest inhibition of caspase-3 activation upon colostrum treatment was shown at 400 μM DEX as 47.5%.

### 2.5. Cell Model of Apoptosis by MC3T3-E1 Cell Exposure to Dexamethasone (DEX)

**Figure 5:**
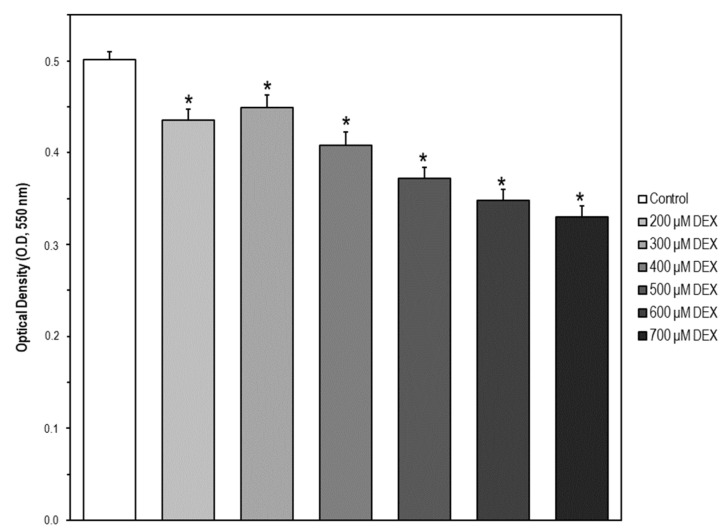
Cell model of apoptosis by MC3T3-E1 cell exposure to dexamethasone (DEX). Cells were treated with DEX in a concentration range of 0–700 μM for 24 h. Then, cell viability was measured by the MTT assay. The data are expressed as the mean ± S.E.M. of three independent experiments. Significant differences were found in optical density (OD, 550 nm) values elicited by every DEX concentration compared to the control untreated cells (Newman–Keuls test, * *p* < 0.05 vs. control).

MC3T3-E1 cells were treated with DEX in a concentration range of 0–700 μM. Using the MTT assay we observed that the cell viability was significantly decreased in a concentration-dependent manner (Figure 5). The concentrations of 200, 400, and 700 μM were chosen for the subsequent experiments as low, medium, and high DEX concentrations, respectively.

### 2.6. Suitable Concentration Range of Colostrum Which Is Non-Cytotoxic for MC3T3-E1 Cells

**Figure 6:**
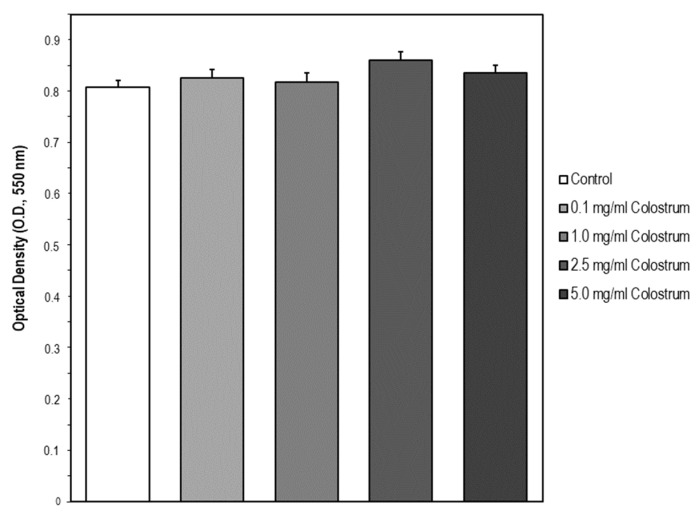
Suitable concentration range of colostrum which is non-cytotoxic for MC3T3-E1 cells. Osteoblast MC3T3-E1 cells were treated with Colostrum (0.1, 1.0, 2.5, and 5.0 mg/mL) for 24 h. Then, cell viability was measured by the MTT assay. The data are expressed as the mean ± S.E.M. of three independent experiments. No significant differences were found in OD values elicited by every Colostrum concentration compared to the control untreated cells (Newman–Keuls test).

The second step aimed at identifying a suitable concentration range of bovine colostrum for further assessing its potential protective action in a cell model of DEX-treated osteoblast MC3T3-E1. The concentrations selected were taken from Mussano et al. [34] and were 0.1, 1.0, 2.5, and 5 mg/mL of culture media. After colostrum treatment for 24 h, cell viability was assessed using the MTT assay. Cell viability was not affected by the bovine colostrum compared to untreated control cells, meaning, it was not cytotoxic for MC3T3-E1 cells at the concentration range tested (Figure 6).

### 2.7. Colostrum Rescues MC3T3-E1 Cells from DEX Cytotoxicity

**Figure 7:**
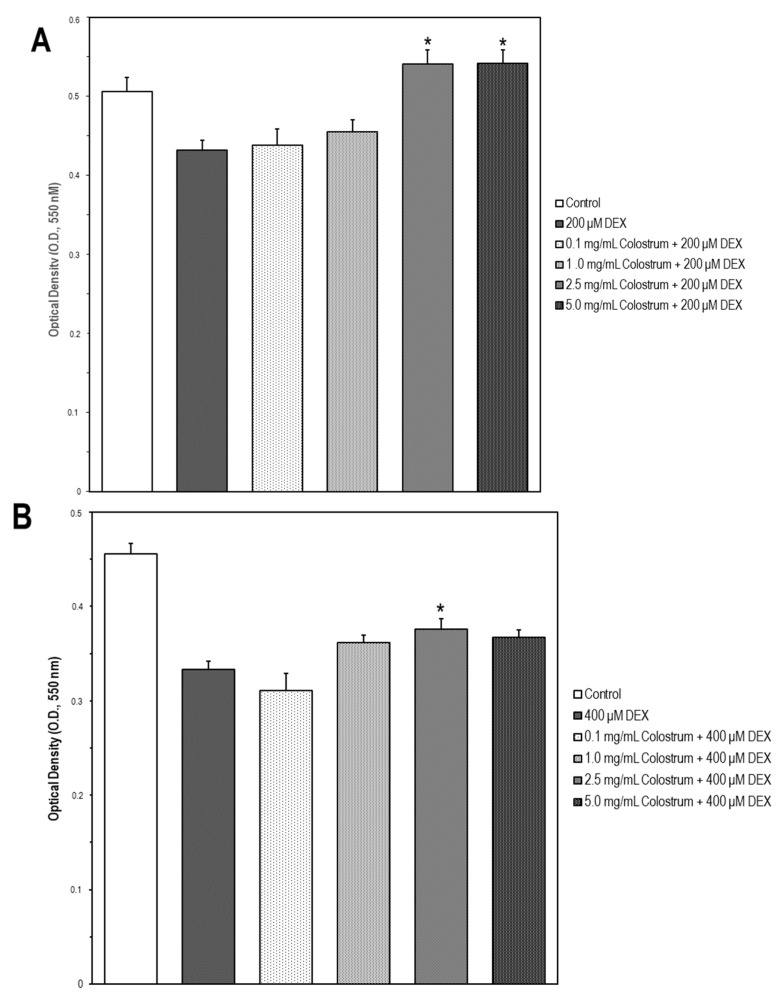

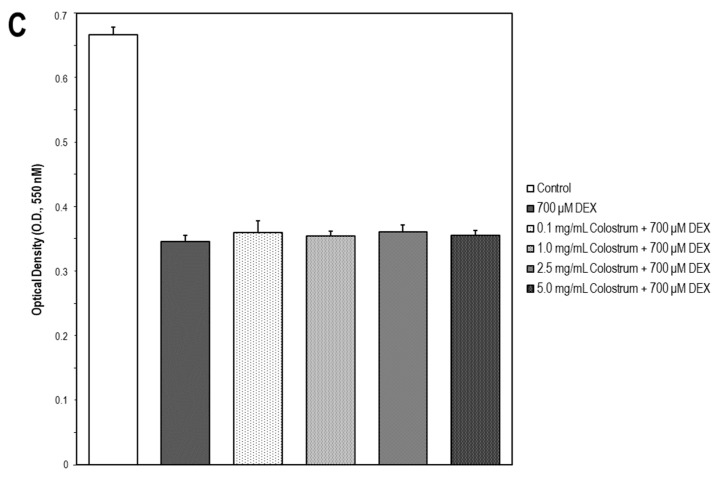
Colostrum rescues MC3T3-E1 cells from DEX cytotoxicity. MC3T3-E1 cells were treated with colostrum (0.1, 1.0, 2.5 and 5.0 mg/mL) for 1 h and then cell incubation continued with DEX (200, 400, or 700 μM) for 24 h. Cell viability was measured by the MTT assay. The data are expressed as the mean ± S.E.M. of three independent experiments. (**A**) Significant increases were found in OD values elicited by 2.5 or 5 mg/mL colostrum + 200 μM DEX compared with the 200 μM DEX treated cells (Newman–Keuls test, * *p* < 0.01 vs. 200 μM DEX cells). (**B**) Significant differences in OD values were observed in 2.5 mg/mL colostrum + 400 μM DEX compared to the 400 μM DEX treated cells (Newman–Keuls test, * *p* < 0.05 vs. 400 μM DEX cells). (**C**) No significant differences were found in OD values displayed by any colostrum concentration + 700 μM DEX compared to the 700 μM DEX treated cells.

To investigate whether the bovine colostrum may protect MC3T3-E1 cells against DEX-induced cytotoxicity, the cells were treated with the different concentrations of colostrum (0.1, 1.0, 2.5, and 5.0 mg/mL) for 1 h. Then, DEX was added to the culture media at 200, 400, and 700 μM, respectively, and cell incubation continued for 24 h. Afterwards, cell viability was examined, and it was found that colostrum was more capable of rescuing cells in the presence of 200 μM DEX (Figure 7A) than in that of 400 μM DEX (Figure 7B). However, no changes were found in cell survival of 700 μM DEX-treated cells upon colostrum treatment (Figure 7C).

### 2.8. DEX-Induced Caspase-3 Activation Is Attenuated by Colostrum Treatment

**Figure 8:**
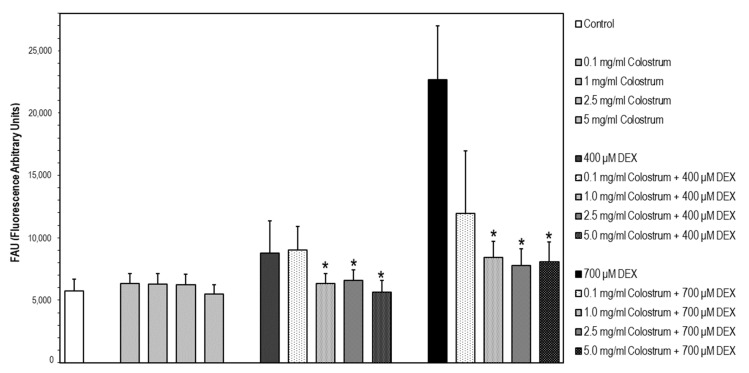
DEX-induced caspase-3 activation is attenuated by colostrum treatment. Caspase-3 activity was determined using the fluorogenic substrate Ac-DEVD-AMC. Data are expressed in fluorescence arbitrary units (FAU) and represent the mean ± S.E.M. of three independent experiments. * Significant differences of colostrum plus 400 μM DEX or colostrum plus 700 μM DEX treated cells versus DEX-treated cells (Newman–Keuls test, * *p* < 0.05 vs. DEX treated cells).

It has been described that DEX induces apoptosis in the MC3T3-E1 osteoblastic cell line as a consequence of activation of caspase-3, which is a general downstream effector of various apoptotic signaling pathways [35]. In our experiments, the lowest DEX concentration that rendered a significant increase in caspase-3 activation versus untreated cells was 400 μM. In the first step, cells were treated with the different concentrations of colostrum (0.1, 1.0, 2.5, and 5.0 mg/mL) for 1 h, and then, DEX was added to the culture media at 400 or 700 μM, and cell incubation continued for 24 h. Afterwards, caspase-3 activation was determined. For both DEX concentrations, colostrum was capable of attenuating caspase-3 activation from the concentration of 1.0 mg/mL (Figure 8). The colostrum did not affect caspase-3 activation.

### 2.9. Colostrum Treatment Restores GSH Levels in DEX-Treated MC3T3-E1 Cells

**Figure 9:**
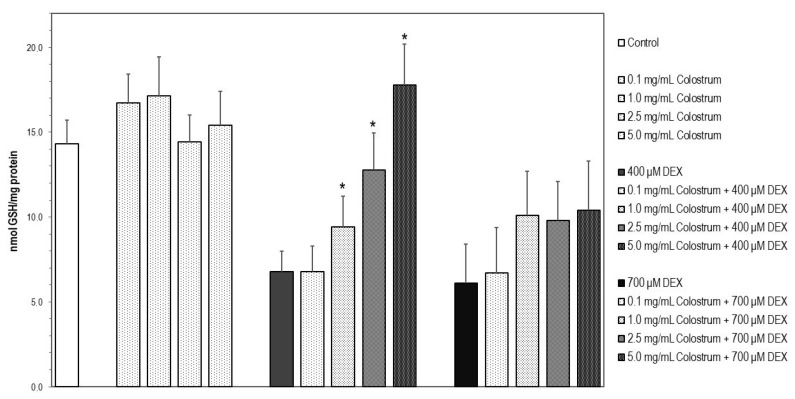
Colostrum treatment restores GSH levels in DEX-treated MC3T3-E1 cells. Reduced glutathione (GSH) was determined spectrofluorometrically [36]. Cells were treated with quercetin or rutin for 24 h. The concentration of GSH in each sample was interpolated from known GSH standards. Fluorescence was measured at λexc = 350 nm (bandwidth 5 nm) and λem = 420 nm (bandwidth 5 nm). Protein was measured by the bicinchoninic acid method. Values were expressed as nmol/mg of protein. * Significant differences of colostrum plus 400 μM DEX versus 400 μM DEX-treated cells (Newman–Keuls test, * *p* < 0.05).

Cells upon DEX treatment with 400 or 700 μM for 24 h underwent a significant and similar reduction in their GSH levels in comparison with untreated control cells (Figure 9, *p* < 0.05). The decrease in GSH content at 400 μM DEX cells was significantly prevented by 2.5 and 5 mg/mL colostrum treatment (*p* < 0.05) since the redox status of GSH achieved was found to be similar to that of detected in control MC3T3-E1 cells (Figure 9). Increases in GSH levels observed in 700 μM DEX-treated cells upon colostrum exposure were not significantly different when compared with 700 μM DEX-treated cells and were non-concentration dependent (Figure 9).

### 2.10. Colostrum Treatment Increases p-ERK1/2 Levels in DEX-Treated MC3T3-E1 Cells

**Figure 10:**
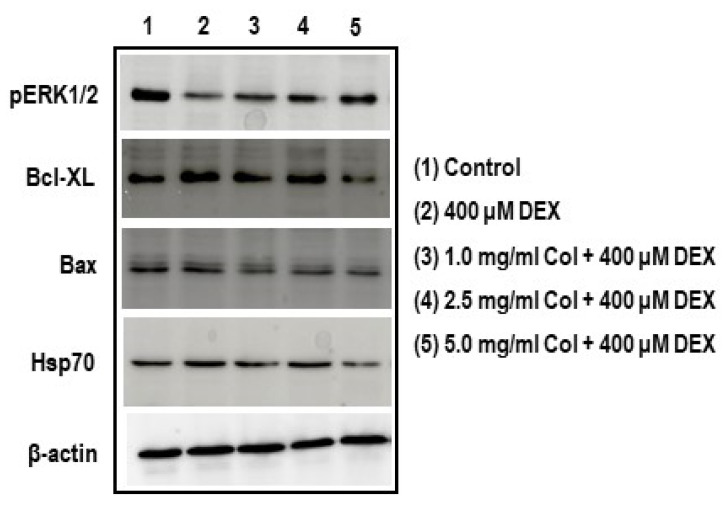

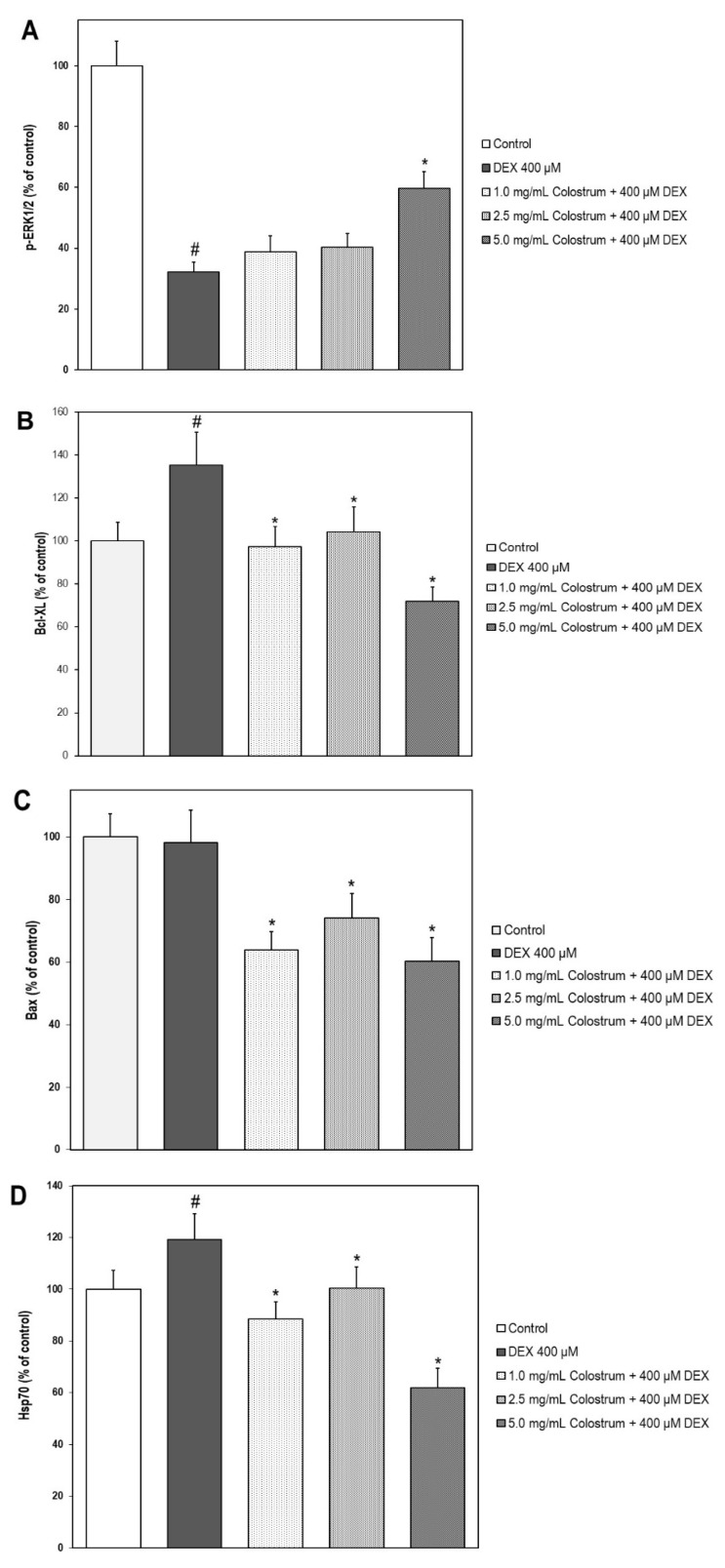
Effect of colostrum treatment on p-ERK1/2, Bcl-XL, Bax, and Hsp70 protein levels on DEX-treated MC3T3-E1 cells. Phospho-ERK1/2, Bcl-XL, Bax and Hsp70 protein levels were detected by Western blot analysis. The levels of the p-ERK1/2 (**A**), Bcl-XL (**B**), Bax (**C**), and Hsp70 (**D**) were represented as relative percentages of the control after its normalization by the β-actin. The data are expressed as the mean ± S.E.M. of three independent experiments. * Significant differences of colostrum plus 400 μM DEX versus 400 μM DEX-treated cells (Newman–Keuls test, * *p* < 0.05). ^#^ Significant differences of 400 μM DEX-treated cells versus control cells (Newman–Keuls test, ^#^ *p* < 0.05).

Cells, upon DEX treatment with 400 μM for 24 h, experienced a significant reduction in p-ERK1/2 levels in comparison with untreated control cells (Figure 10A, *p* < 0.05). The decrease in p-ERK1/2 levels at 400 μM DEX cells was partially alleviated by 5.0 mg/mL colostrum treatment (*p* < 0.05) (Figure 10A, *p* < 0.05).

### 2.11. Colostrum Treatment Diminishes Bcl-XL Levels in DEX-Treated MC3T3-E1 Cells

A significant augmentation in Bcl-XL levels in comparison with untreated control cells was observed on cells upon DEX treatment with 400 μM for 24 h (Figure 10B, *p* < 0.05). The increase in Bcl-XL levels at 400 μM DEX cells was returned to control levels by 1.0 and 2.5 mg/mL colostrum treatment (Figure 10B, *p* < 0.05).

### 2.12. Colostrum Treatment Diminishes Bax Levels in DEX-Treated MC3T3-E1 Cells

Unchanged levels of Bax were found in cells upon DEX treatment with 400 μM for 24 h when compared with those of untreated control cells (Figure 10C). However, significant reductions in Bax levels were observed in DEX-treated cells when they were exposed to any colostrum concentration (Figure 10C, *p* < 0.05). No significant differences were found in Bax levels among the three colostrum concentration treatments (Figure 10C).

### 2.13. Colostrum Treatment Attenuates Hsp70 Levels in DEX-Treated MC3T3-E1 Cells

Cells, upon DEX treatment with 400 μM for 24 h, experienced a significant increase in Hsp70 levels in comparison with untreated control cells (Figure 10D, *p* < 0.05). The increase in Hsp70 levels at 400 μM DEX cells was returned to control levels by 1.0 and 2.5 mg/mL colostrum treatment (Figure 10D, *p* < 0.05) and even lower by 5.0 mg/mL (Figure 10D, *p* < 0.05).

## 3. Discussion

Based on the fact that high doses of dexamethasone (DEX) induce neurodegeneration and that glucocorticoid-induced osteoporosis (GIO) is one of the most common secondary and iatrogenic forms of osteoporosis, the present study hypothesized that neuroprotective bovine colostrum against DEX-induced neuronal damage may exert protective effects against glucocorticoid-induced osteoblast injury. 

Glucocorticoid-induced damage in the SH-SY5Y human neuroblastoma cell line was generated by long-term exposure to DEX. Previous studies have shown that cell exposure to DEX for 24 h causes significant inhibition of the PI3K/Akt signaling pathway enhancing DEX-induced cell death [37]. Neurotoxicity upon DEX treatment for 48 h was shown by the lowering of the cell viability, increasing in intracellular ROS generation, and caspase-3 activation. Under the experimental conditions in our study, the SH-SY5Y cells, co-treated with the bovine colostrum and DEX for 48 h, experienced an increase in cell viability in a concentration-dependent manner. An intermediate concentration of the bovine colostrum (1.0 mg/mL) was able to rescue cells by scavenging intracellular ROS and attenuating caspase-3 activation. Compounds attenuating DEX-induced reduction in SH-SY5Y cell viability and proliferation have demonstrated an involvement of the activation of the mitogen-activated protein kinase (MAPK)/ERK1/2 pathway [38]. Interestingly, among the active constituents of colostrum that have been highlighted in the introduction section, it is worth mentioning here the IGF-1 as a putative neuroprotective growth factor for the neuronal cell model of this study since it exerts its biological function by binding to IGF-1 receptor and subsequently activates both PI3K/Akt and MAPK/ERK signal pathways protecting SH-SY5Y cells from apoptosis [39]. Another remarkable component of bovine colostrum for its reported neuroprotective activity is colostrinin. It is incompletely defined chemically and largely consists of a mixture of at least 32 peptides (proline-rich polypeptides, PRPs) whose biological activity seems to be based on more than one component [40]. This colostral PRP complex has been shown in cultured cells that modulates intracellular ROS levels, via regulation of glutathione metabolism, activity of antioxidant enzymes, and mitochondria function [41]. Therefore, based on these neuroprotective mechanisms, it could be suggested that apoptosis in neuron cells derived from high DEX levels which lead to increases in ROS production directly causing mitochondria dysfunction and caspase-3 activation [42] may be attenuated by the PRP component of the colostrum. 

As for the osteoblastic cellular system upon this study, glucocorticoid-induced injury was initiated in the MC3T3-E1 murine osteoblastic cell line by exposure to DEX. The results demonstrated that exposure of MC3T3-E1 cells to DEX induced cytotoxicity, as determined by decreased cell viability and intracellular GSH levels and increased caspase-3 activation. To investigate whether the colostrum may protect MC3T3-E1 cells against DEX-induced cytotoxicity, cells were treated with colostrum at concentrations ranging between 0.1 and 5.0 mg/mL for 1 h prior to DEX exposure and for an additional period of 24 h in co-treatment with DEX.

Increased oxidative stress is currently considered a crucial cause of GIO. This study showed that DEX raised cellular oxidative stress, as evident with the decay of GSH levels, and simultaneously caused reductions in cell survival, and initiation of cell apoptosis as shown by caspase-3 activation. It is possible that the protective effect of the colostrum against DEX dysfunction could be due to its ability to modulate intracellular GSH levels, which is in agreement with the activities of the abundant antioxidant compounds occurring in bovine colostrum. For instance, lactoferrin alleviates aging-related gene expression, improves antioxidant enzyme activity, and reduces oxidative injury in vitro through IGF-1 in senescent osteoblasts [43]. As for the PRP complex, the left-handed-polyproline-II helix of its structure has shown to be essential for the antioxidant properties and finely modulation of protein–protein interactions [44]. 

It is interesting to note that DEX exposure induced Bcl-XL in the MC3T3-E1 osteoblastic cells, which indicates that DEX may act as an anti-apoptotic factor. Alternatively, Bcl-XL may be cleaved by caspase-3, resulting in a potent apoptosis inducer [45]. This means that cleavage of Bcl-XL during the execution phase of cell death converts Bcl-XL from a protective to a lethal protein. In our study, since caspase-3 activation was triggered by DEX, it is likely that augmenting Bcl-XL levels places the osteoblasts in a vulnerable condition that could be overcome by colostrum treatment, as Bcl-XL levels were lowered. 

The role of the Bax protein, one of the Bcl-2 family members, in the apoptosis and cellular proliferation associated with GIO has been previously described [46]. It has been also suggested that the rise of the Bax would trigger cytochrome c release from mitochondria to the cytoplasm, and then activated caspase-9 and caspase-3, thus leading to apoptosis [47]. Western blot results from our experiments clearly showed that the protein expression of Bax was decreased in DEX-treated MC3T3-E1 cells upon colostrum treatment. This implies that the protective effect of colostrum against DEX-induced osteoblast apoptosis is partially mediated by the inhibition of the mitochondrial apoptosis pathway [48].

We have shown thus far that treating the cultured osteoblasts with colostrum elicited a down-regulation of both Bcl-XL and Bax levels as well as an attenuation of caspase-3 activation in DEX-induced apoptotic osteoblasts. 

To this, it should be added that colostrum treatment up-regulated the expression level of p-ERK1/2 in DEX-induced osteoblasts, suggesting that it could activate extracellular signal-regulated kinase (ERK) signaling. Previous studies have revealed that the ERK signaling pathway plays an important role in the regulation of osteogenesis by enhancing osteogenic transcription regulators [49]. It has been also reported that ERK activation can attenuate osteoblast apoptosis induced by DEX [50]. Therefore, our data could suggest that colostrum treatment triggers the activation of the ERK1/2 pathway, inducing an anti-apoptotic effect in osteoblasts, which is consistent with the abundant occurrence of IGF-1 and other growth factors such as PDGF and insulin, in bovine colostrum. IGF-1 is a small polypeptide with homology to pro-insulin that signals via the type 1 IGF-1 receptor (IGF-1R), engaging ERK and phosphatidylinositol 3-kinase pathways through Src homology 2 domain-containing proteins and insulin receptor substrates 1 and 2. The effects of IGF-1 on bone have been well documented showing to induce proliferation of MC3T3 osteoblast-like cells [51]. An analysis of cell cycle distribution in PDGF-treated osteoblasts using flow cytometry has revealed that PDGF treatment increased the number of cells in the S phase and decreased the cell numbers in the G0/G1 phase, indicating that PDGF promoted cell entry into the S phase in osteoblasts and inhibited cell apoptosis [52] being both ERK1 and ERK2 signaling pathways activated by PDGF [53]. Moreover, the ERK pathway has shown to be upregulated in response to insulin treatment in osteoblast-like cells providing that this growth factor is a potent stimulator in survival signaling pathways in insulin-sensitive cells [54].

It is generally recognized that osteoporosis is a common effect found in patients with glucocorticoid excess, and that glucocorticoid receptor is associated with heat shock protein (Hsp) 70 and Hsp90 in a heterocomplex [55]. Hsp70 is transiently induced in response to stress and then rapidly degraded by the proteasome system [56,57]. In fact, we found that osteoblast MC3T3-E1 cells, upon DEX exposure, experienced a significant increase in Hsp70 levels, which returned to control levels and even lower by colostrum treatment. Thus, the objective of a potential colostrum administration as a pharmacological intervention for GIO will be to restore balance to the Hsp70 system in bone. The significance of what has occurred after colostrum treatment of MC3T3-E1 cells on Hsp70 levels could be the result of a modulation of the Hsp70 system. The effect of bovine colostrum on the Hsp70 system has been also studied in human colon cell lines upon temperature-induced apoptosis [58]. In agreement with our data, the colostrum decreased both the pro-apoptotic protein Bax and the activation of caspase-3. However, in contrast to our study, Hsp70 expression was upregulated, which might be related to the type of cell and stimuli causing apoptosis. Hsp70 is present in all of the subcellular compartments of nucleated cells including cell membranes and, as an angiogenesis regulator, Hsp70 can activate ERK [59]. Hsp70 overexpression is associated with inhibition of apoptosis and tumor invasion, on the contrary, downregulation of Hsp70 leads to the demise of cancer cells [60]. With this in mind, it could be assumed that the modulation of the Hsp70 system found in DEX-treated MC3T3-E1 cells by returning to control levels upon colostrum exposure may constitute a mechanism for balancing proliferation and differentiation.

In SH-SY5Y cells upon DEX exposure, increases in intracellular ROS generation were observed together with a lowering in the cell viability and an increase in caspase-3 activation, revealing a potential neuroprotective capability of colostrum against stress hormone-induced cell death via caspase-dependent death processes. However, DEX treatment did not show any significant modification in intracellular ROS generation of osteoblasts in comparison with control cells (data not shown), in contrast to other studies [61] even though caspase-3 activation took place. However, an interesting study performed in MC3T3-E1 cells found a significant increase in ROS production after a period of 3 h DEX incubation [62]. We, therefore, propose that the peak of increased ROS production upon DEX treatment may have occurred at an early stage of incubation with DEX. Furthermore, in terms of cell proliferation/differentiation derived from colostrum cell exposure, either cellular model (SH-SY5Y or MC3T3-E1) upon DEX exposure has been immersed in a mixture of numerous growth factors and cytokines occurring in the bovine colostrum. Since growth factors and cytokines could activate multiple pathways, it is necessary to determine which pathway(s) plays a dominant role in regulating cell proliferation/differentiation. The best-studied ERK activators in osteoblast function are fibroblast growth factors (FGFs) and IGFs [63]. The relative strengths of ERK and Akt signaling pathways determine whether osteoblasts are driven into proliferation or differentiation. Signaling by IGF-1 is known to promote osteoblast differentiation and strongly activates Akt. FGF signaling inhibits differentiation and causes a strong and sustained ERK1/2 activation. Since osteoblast proliferation is one of the most important indicators of osteogenic effect, it was decided to first observe the effect of colostrum on cell proliferation of the MC3T3-E1 cells and the measurement of ERK levels. In addition, alkaline phosphatase (ALP) levels (in a pilot study) were determined as one of the early markers of osteoblast differentiation, showing a significant increase in both 2.5 and 5.0 mg/mL colostrum dosages in comparison with control cells. Then, we propose that both proliferation and differentiation take place and this could explain the weak changes in cell density observed in MC3T3-E1 upon colostrum exposure. As for the neuroblastoma SH-SY5Y cell line, it has been reported that basic FGF (bFGF) and IGF-1 are the two most potent mitogens. However, a combination of bFGF plus IGF-1 has been shown to induce differentiation as bFGF and IGF-1-treated SH-SY5Y cells retained their capacity to proliferate [64]. Thus, we suggest that proliferation in SH-SY5Y cells is more prominent than differentiation and this could explain the higher enhancement in cell density observed upon colostrum cell exposure and the rescue of DEX-injured cell growth at the three DEX concentrations used, in comparison with the osteoblastic cells. This phenomenon seems to be consistent with the abundant occurrence of IGF-1 in bovine colostrum.

Furthermore, the present study provides novel insights into the roles of bovine colostrum in attenuating GIO. We have shown that DEX raised cellular oxidative stress and simultaneously caused reductions in cell survival, and induction of cell apoptosis. We have also demonstrated that colostrum could ameliorate GIO by protecting osteoblasts from apoptosis, possibly via the activation of the ERK pathway and modulation of the Hsp70 system. 

Our work reported here was designed to ascertain whether a neuroprotective colostrum against DEX neuronal damage has any effects on DEX-induced osteoblast damage. We provided preliminary evidence on how this colostrum, as a complex and multi-component dairy product, may affect osteoblastic cell survival undergoing apoptosis in addition to its neuroprotective action. Then, we suggest that the present bovine colostrum possesses potential as a natural resource for the prevention and/or treatment of two closely disorders in postmenopausal women, osteoporosis, and impairment of the nervous system, caused by chronic dexamethasone treatment. However, as in vitro models lack the complexity of entire systems, our findings should be further corroborated using in vivo models.

## 4. Materials and Methods

### 4.1. Bovine Colostrum

Bovine colostrum was obtained from a local milk producer. As bovine colostrum can be an important vector for many important disease-causing pathogens, such as *Mycobacterium avium paratuberculosis*, *Salmonella*, *Mycoplasma*, *Listeria*, *E. coli*, and many others, it was important to follow the Guidelines from the European Commission (Regulation No 1662/2006 and No 1663/2006) in order to obtain pathogen-free bovine colostrum for cell supplementation. Specifically, the following steps were taken to prepare bovine colostrum for cell supplementation: (1) bovine colostrum was collected from cows (4–8 h after giving birth); (2) a lyophilization process was initiated; and (3) a process of pasteurization was initiated involving exposure to high temperature (72 °C) for 15 s, followed by low temperature (63 °C) for 30 min. 

### 4.2. Culture of the Human Neuroblastoma SH-SY5Y Cells

Human neuroblastoma (SH-SY5Y) cells were originally derived from the SK-N-SH cell line. The SH-SY5Y cells were maintained in Dulbecco’s Modified Eagle’s Medium (DMEM) (Lonza BioWhittaker, Porriño, Spain) supplemented with 10% fetal bovine serum (Lonza BioWhittaker, Spain) and 50 μg/mL gentamycin (Gibco, Madrid, Spain) and maintained at 37 °C in a humidified atmosphere of 5% CO_2_. 

### 4.3. Culture of the Mouse MC3T3-E1 Osteoblast Cell Line

The mouse MC3T3-E1 osteoblast cell line was kindly donated by Dr. Isabel Izquierdo-Barba (Departamento de Química en Ciencias Farmacéuticas, Universidad Complutense de Madrid. Instituto de Investigación Sanitaria Hospital 12 October i + 12, Madrid, Spain). The cells were cultured in α-minimum essential medium (α-MEM) without L-ascorbic acid (Lonza BioWhittaker, Porriño, Spain) supplemented with 10% fetal bovine serum (FBS) (Lonza BioWhittaker, Spain), 2 mM glutamine and 50 μg/mL gentamycin (Gibco, Spain) and maintained at 37 °C in a humidified atmosphere of 5% CO_2_. 

### 4.4. Cell Treatment

During the logarithmic growth phase, cells were digested with 0.25% trypsin and then suspended in culture media. The cell suspension was centrifuged gently (100× *g*, 5 min, RT) and the trypsin-containing medium was removed. Fresh medium was added to cells and they were seeded at appropriate densities on plates according to each experimental scale. Experiments were always carried out 24 h after cells were seeded. For treatments, SH-SY5Y or MC3T3-E1 cells were incubated with DEX for 24 h, or with colostrum for 1 h plus an additional period of 24 h in co-treatment with DEX in culture media containing 1.0% FBS. The concentrations tested of the colostrum (0.1, 1.0, 2.5, and 5.0 mg/mL) were based on the work by Mussano et al. [33]. Particularly, in order to stimulate apoptosis in the osteoblast cultures, cells were exposed to DEX at concentrations upon which proliferation and differentiation are maximally inhibited and, specifically, right after cells have reached the stage of confluency [65]. Research on the biocompatibility of biomaterials for bone regeneration has provided us a good basis for the borderline DEX dose (toxicity-free dose) and the optimization of the DEX concentration range that triggers apoptosis in MC3T3-E1 cultures [66]. Accordingly, both lower and higher concentrations than 500 μM were tested.

### 4.5. Cell Viability Assay

Cells were plated in 96-well polystyrene plates with 20,000 cells per well (SH-SY5Y) or 10,000 cells per well (MC3T3-E1) and incubated at 37 °C for 24 h to allow cells to attach. The plates were then incubated with DEX or colostrum or DEX plus colostrum 24 h. Cell viability was quantified by measuring the metabolic activity by the mitochondrial-dependent reduction of MTT (3-(4,5-dimethylthiazol-2-yl)-2,5-diphenyltetrazolium bromide (Sigma-Aldrich) to its insoluble formazan [67]. The absorbance was read at 550 nm using a SPECTROstar microplate reader (BMG LABTECH).

### 4.6. Reactive Oxygen Species (ROS) Measurement in SH-SY5Y Cells

The molecular probe dichlorofluorescein diacetate (DCFA-DA) was used to measure intracellular ROS generation in SH-SY5Y cells. For this assay, cells were subcultured, and 24 h later they were loaded with 10 μM DCFA-DA, which diffuses through the cell membrane and is hydrolyzed by intracellular esterases to the dichlorofluorescein (DCFH). DCFH reacts with intracellular free radicals to form dichlorofluorescin (DCF), a green fluorescent dye. Colostrum was added to the cells 60 min prior to the treatment with DEX which was maintained for 48 h on culture media. The fluorescence caused after exposure of the cells to colostrum plus DEX was measured with a FLUOSTAR microplate reader (BMG LABTECH) with the excitation filter set at 485 nm (bandwidth 5 nm) and the emission filter set at 520 nm (bandwidth 5 nm). The data were presented as fluorescence arbitrary units (FAU). 

### 4.7. Determination of Caspase-3 Activity

Caspase-3 activity assay was conducted using a fluorogenic substrate Ac-DEVD-AMC for caspase-3 (Sigma-Aldrich, St. Louis, MO, USA). Cells were collected, washed with ice-cold PBS, and lysed with 100 µL of lysis buffer (50 mM HEPES, pH 7.4, 100 mM NaCl, 0.1% CHAPS, 10% glycerol, 10 mM dithiothreitol and 0.1 mM EDTA). After the cells were treated as indicated in *cell treatment,* cell lysates were collected, sonicated, and centrifuged at 14,000× *g* for 15 min at 4 °C. Briefly, 20 µg of cellular protein (from whole-cell lysates) was incubated with 20 µM caspase-3 substrate (Ac-DEVD-AMC) in a buffer consisting of 50 mM HEPES (pH 7.4) with 100 mM NaCl, 0.1% CHAPS, 10% glycerol, and 10 mM dithiothreitol, for 2 h at 37 °C. Substrate cleavage was measured with a FLUOSTAR microplate reader (BMG LABTECH) with the excitation filter set at 360 nm and the emission filter set at 460 nm. The activity of caspase was calculated from the cleavage of the specific fluorogenic substrate. The data were presented as fluorescence arbitrary units (FAU).

### 4.8. Glutathione Content

Reduced glutathione (GSH) was determined spectrofluorometrically [35]. Cells were treated as indicated in cell treatment. The fluorophore o-phthalaldehyde (OPA) was used as a derivatizing agent. OPA is non-fluorescent until it reacts with a primary amine in the presence of thiol, cyanide, or sulfite, forming a fluorescent isoindole. The following solutions were required to perform the OPA assay: redox quenching buffer (RQB) (20 mM HCl, 5 mM DTPA, 10 mM ascorbic acid); 5% TCA in RQB (TCA–RQB); 7.5 mM N-ethylmaleimide (NEM) in RQB; 100 mM dithionite (DT; sodium hydrosulfite) in RQB; 5.0 mg/mL OPA in methanol. Cell supernatant samples were deproteinized in TCA–RQB. To estimate the background fluorescence of a sample, NEM was added to sequester the GSH from it. The assay consisted of paired samples, labeled A and B. Sample A was the background consisting of non-GSH-dependent fluorescence that was subtracted from the paired sample B. GSH levels were determined using 10 μL of deproteinized cell supernatant which was incubated with OPA for 30 min at room temperature. The concentration of GSH in each sample was interpolated from known GSH standards. OPA-derived fluorescence was measured at λexc = 350 nm (bandwidth 5 nm) and λem = 420 nm (bandwidth 5 nm) with a FLUOSTAR microplate reader (BMG LABTECH). Protein was measured by the bicinchoninic acid method. Values were expressed as nmol/mg of protein.

### 4.9. Analysis of pERK1/2, Bcl-XL, Bax and Hsp70 by Western Blot

After cells were treated as indicated in cell treatment, they were harvested, washed twice with PBS, and recovered by centrifugation at 1000× *g* for 5 min. The cell pellet was resuspended in PBS and centrifuged at 100× *g* for 10 min at 4 °C to remove the supernatant. After centrifugation, cell pellet samples were lysed and sonicated in a lysis buffer consisting of 10 mM Tris-HCl (pH 7.5) with 0.5% CHAPS, 1 mM Cl_2_Mg, 1 mM EGTA, 1 mM EDTA, 10% glycerol, 5 mM β-mercaptoethanol, 1 mM DTT, 1 mM phenylmethylsulfonyl fluoride, 100 µM leupeptin, and 1 µM pepstatin. After incubation for 30 min on ice, the cell lysate was cleared by centrifugation at 13,000× *g* for 5 min. Protein determinations were performed in the supernatant according to the bicinchoninic acid method. An equal amount of protein from each cell lysate sample was separated in 7.5% SDS-PAGE minigels and electroblotted to PVDF membranes in a buffer containing 0.025 M Tris-HCl, 0.192 M glycine, and pH 8.3 at 350 mA for 1 h. After blocking with 10% non-fat milk (or 5% BSA for phosphorylated antibodies), immunostaining reaction was performed, respectively, with a polyclonal anti-p-ERK1/2 (Phospho-Thr202/Tyr204) (GenScript, 1:1000), a polyclonal anti-Bcl-XL (Santa Cruz (S-18) (SC634), 1:1000), a monoclonal anti-Bax (Sigma B-8429, 1:5000), a monoclonal anti-Hsp70 (Sigma H-5147, 1:100,000) and a monoclonal anti-β-actin (Sigma A-5441, 1:100,000) as the internal standard. After an overnight incubation, blots were incubated for 1 h with a peroxidase-conjugated secondary antibody. This was followed by detecting the chemiluminescence of the binding by means of visualizing equipment (ImageQuant™ LAS 500). The intensity of the bands was determined using the software ImageQuant TL 8.2 and normalized to the bands of the internal standard (β-actin).

### 4.10. Statistical Analyses

All experiments were conducted in at least three independent experiments and all assays were prepared in triplicates. Data were analyzed using a one-way analysis of variance (ANOVA) followed by a Newman–Keuls test. Statistical significance for all parameters was set at *p* < 0.05.

## Data Availability

Data is contained within the article.

## Abbreviations

DEX	Dexamethasone
FAU	Fluorescence arbitrary units
FBS	Fetal bovine serum
GIO	Glucocorticoid-induced osteoporosis
GSH	Glutathione reduced
MTT	3-[4,5-dimethylthiazol-2-yl]-2,5-diphenyltetrazolium bromide
OD	Optical density
ROS	Reactive oxygen species
